# Exploring different stakeholders’ perspectives on ward rounds in paediatric oncology: a qualitative study

**DOI:** 10.1186/s12909-023-04447-2

**Published:** 2023-07-06

**Authors:** Lea P. Berndt, Julia Sellin, Urs Mücke, Martin Mücke, Rupert Conrad, Lorenz Grigull

**Affiliations:** 1 Department of Paediatric Oncology, Hannover Medical School, Hannover, Germany; 2 Institute for Digitalization and General Medicine, University Hospital RWTH Aachen, Aachen, Germany; 3 Department of Psychosomatic Medicine and Psychotherapy, University Hospital Muenster, Muenster, Germany; 4 University Hospital Bonn, Centre for Rare Diseases, Bonn, Germany

**Keywords:** Ward round, Insight, Interview, Multidisciplinary, Family-centred, Team

## Abstract

**Rational/Aims and Objectives:**

Ward rounds are a core routine for interprofessional communication and clinical care planning: Health care professionals and patients meet regularly and it encourages patients to actively participate. In paediatric oncology, the long treatment process, the serious diagnosis, and involvement of both patients and their parents in shared-decision-making require specific ward round skills. Despite its high value for patient-centred care, a universal definition of ward round is lacking. Little is known about attitudes and expectations of different participants towards a ‘good’ ward round. This study aims to capture experiences and expectations of different stakeholders to better understand ward round needs in paediatric oncology and serve as a basis to improve future ward rounds.

**Method:**

Semi-structured interviews were conducted with patients, parents, nurses and medical doctors of a paediatric oncology ward until theoretical saturation (13 interviews). A standardised qualitative analysis using the phenomenological framework defined by Colaizzi was used to identify important aspects in the interviews.

**Results:**

Three major themes were identified in the interviews: [1] Structure and Organisation; [2] Communication; [3] Education. Further analysis revealed 23 categories and elucidated several opportunities and unmet needs recognized by stakeholders: Ward round functions in comforting families in stressful situations, and relationship building. Interviewees expressed their concerns about missing structures. Families pleaded for smaller ward round teams and layperson language. Health care professionals underscored the lack of ward round training. Paediatric patients stated that ward round scared them without proper explanation. All interviewees emphasized the need for professionalization of the ward round in the setting of paediatric oncology.

**Conclusion:**

This study gives important insights into ward round functions and organisational requirements. It addresses special challenges for ward round participants in paediatric oncology, such as consideration of the emotional aspect of cancer treatment or the limits of shared decision making. Furthermore, this study underscores the great significance of ward rounds in paediatric oncology, with an emphasis on communication and relationship-building. Although performed universally, ward rounds are poorly explored or evaluated. This structured analysis synthesizes important expectations of different WR stakeholders, revealing opportunities of improvement and stressing the need for guidelines, training, and preparation.

## Background

In hospital care, the ward round (WR) can be regarded as one essential regular part of clinical hospital practice, where the multidisciplinary team meets to exchange information and discuss further care planning [1, 2]. WR can take place in a conference room, but also at the patient’s bedside, giving patients the opportunity to ask questions and take an active role in their therapy process [3]. With patient centred care and patient autonomy becoming more and more important in modern medical care, bedside rounds represent a cornerstone in the therapeutic process [4]. WR culture, frequency, length and focus differ with clinical specialty and between countries. Despite being a clinical core routine in every hospital ward, the one and only definition of a WR does not exist [5] and research on WR is limited. Performing WR is also not systematically taught at medical or nursing school [6]. Young HCP learn to conduct a WR ‘on the job’, mostly without scientifically proven recommendations or standardised feedback procedures [7]. Recent research shows that the setting, such as timing and location, as well as good preparation, elimination of distraction and clear structures are of great importance for a successful WR. Besides that, teamwork, leadership and communication also have to be taken into consideration for successful WR [1, 8, 9]. While there is a fair amount of research on certain WR aspects, holistic information about participants’ attitudes, personal experiences, and the team’s view on WR are still lacking.

In paediatric oncology, parents usually participate in the WR. Research demonstrates that having parents involved can increase the satisfaction of both health care professionals (HCP) and families [10] and shorten hospital stay [11]. When it comes to shared decision making, there are not only two, but three parties involved in the process. In addition, finding the best strategies on involving underage patients into making decisions is still subject of current research [12, 13]. Therefore while family-centred rounds have become standard practice on many paediatric wards, they also require special communication skills [14] and an additional focus on shared decision making, which makes paediatric WR even more complex. In paediatric oncology, the burden of a cancer diagnosis and the long-lasting therapy process poses significant emotional stress on families, which always has to be considered during WR [15, 16]. Even though there is no research on how long an average paediatric oncology WR takes, considering the complex, long-lasting treatment process, as well as a lot of additional (e.g. psychosocial) information that have to be discussed, it can be assumed that WR requires a relevant proportion of time within the daily routine of an oncology ward.

Conducting a successful multiprofessional WR might be hampered by the lack of a comprehensive understanding of functions, challenges, and chances that WR provides for different stakeholders. All WR participants throughout their work life or treatment process have experienced both successful and failed WR. But when evaluating a WR, participants can only include their own experience. However, a WR can only be considered successful when meeting the expectations of all participants. To gain a holistic understanding of what WR means to these different stakeholders in the setting of a paediatric oncology round, semi-structured interviews with medical doctors, nurses, patients, and parents were conducted. The primary aim was to explore experiences and perceptions made by different participants during WR to synthesize a systemic view on essential WR functions. We expect the results to contribute to a knowledge base for defining a successful WR, to help participants improve their WR skills, and the development of interdisciplinary WR training programs in medical and nursing school.

## Methods

### Design

For this study, face-to-face semi-structured interviews were conducted from January 2018 until August 2019. Interviews were transcribed and analysed using a qualitative descriptive method (Colaizzi[17]), in order to gain a deeper understanding of the lived experience of individuals [18]. Colaizzi’s framework is assigned to descriptive phenomenology, a research method that aims to understand the experienced reality of individuals in complete purity [19]. It enables a clear and structured analysing process, while always staying close to the original text. It also refers clusters of statements back to the original text regularly to make sure that information does not get lost or misinterpreted[20]. Additionally, it provides a realistic understanding of the meaning of what participants stated[21]. As it was not aimed to collect definitions but by contrast to explore observations and experiences regarding ward rounds the question for a definition of ward round was omitted. Possibly, asking interviewees for a ‘clear definition’ might have been interesting but could have resulted in a constricted atmosphere and different direction of the interviews.

The open study design allowed participants to describe their personal perception freely to ensure no important aspects were missed[22]. All methods were carried out in accordance with relevant guidelines and regulations.

### Participant selection

Ethics committee approval was obtained from Ethics committee of Hannover Medical School prior to recruitment (No. 7700, 05.03.2018). All participants and for minors, their legal guardian gave written informed consent before participating in the interviews. All experiments were performed in accordance with relevant guidelines and regulations. All participants were recruited from the paediatric oncology ward by one of the team members (LG). This was done by handing out information leaflets to potential participants and waiting for their answer of assurance. Interviewees’ decision to participate or not was not disclosed to HCP involved in patients’ treatments. Inclusion criteria were: able to speak and read German; participation in > 10 WR as a paediatric (oncology) nurse/assistant physician/patient/parent; minimum age 11 (patients); ability to provide written consent for participation, additionally written consent by a parent for minors. An overview of respondents is provided in Table 1.

**Table 1:** Overview of all interviewees. F = Female, M = Male, HCP = Health care professional, PP = Parent/Patient, AML = Acute myeloid leukemia, ALL = Acute lymphoid leukemia

Interview	Group	Sex	Age (years)	Duration (min)	Work experience (years)	Patient’s disease	Time from Diagnosis
HCP1	Nurse	M	23	27:55	1	-	-
HCP2	Nurse	F	54	30:34	31	-	-
PP1	Parent	F	44	31:14	-	Daughter: ALL	6 months
PP2	Parent	M	41	40:41	-	Daughter: AML	8 months
HCP3	Doctor	F	28	26:09	3	-	-
HCP4	Doctor	F	32	22:02	5,5	-	-
PP3	Patient	M	15	07:03	-	ALL	4 months
HCP5	Doctor	M	28	14:07	2,5	-	-
PP4	Patient	F	17	12:25	-	Lymphoma	3.2 months
HCP6	Nurse	F	31	25:13	11	-	-
PP5	Patient	F	18	32:29	-	AML	9 months
PP6	Parent	M	28	22:59	-	Son: AML	4 months
PP7	Patient	M	11	10:15	-	Medulloblastoma	4 months

### Setting

Information leaflets were handed out to patients and HCP to inform about the study beforehand. Interviews took place in a regular office room of the department of paediatric oncology at Medical School of Hannover (MHH). During two of the four patient’s interviews a parent was present, though not involved in the conversation. Prior to the interview and its recording, a comprehensive written information about the project was handed to all participants. Open questions were settled. Participants (and additionally the parent/legal guardian, when participants were minors) were asked to sign the declaration of consent before the interview.

### Data collection

Interviews were conducted by a medical doctor candidate (LB) with medical background and knowledge, who did not work on the oncology ward, nor had a personal connection to any of the study participants. Interviews started with a uniform beginning (“*As a [nurse/medical doctor/parent/patient] you have already experienced several WR. In your own words, what is the meaning of the WR for you?”).* For comparability, a structured part using an interview guide followed (“*Do you remember the first ward round you attended? In your opinion, what makes a particularly good ward round? On the other hand, what makes a particularly bad ward round? What results do you want to have after a successful ward round? What would a ‘perfect’ ward round be like for you? Could you describe your part or tasks in ward rounds?”* Spontaneous follow-up questions were then asked to gain a deeper understanding of what participants told before. After all open questions were clarified, the final question was: “*Is there anything we have not talked about? Is there any advice you want to give us”?*). The interview guide was developed beforehand, based on literature research with the keywords „ward round“; „paediatric oncology“; „interdisciplinary“; „family centred round“; „teaching“; „medical students“; „communication“; „time“; „decision making”. After reading the retrieved publications, two researchers (LB, LG) formed the framework for the interview guide by discussing relevant ward round aspects encountered in the literature. After consensus was reached with regard to relevant topics, the corresponding questions were formulated as open as possible, to capture divers individual viewpoints and experiences without narrowing down the focus of the interview in advance. A minimum of three interviews per group were planned, but in addition it was agreed to perform interviews until the theoretical saturation was achieved [23].

### Data analysis

**Fig. 1:**
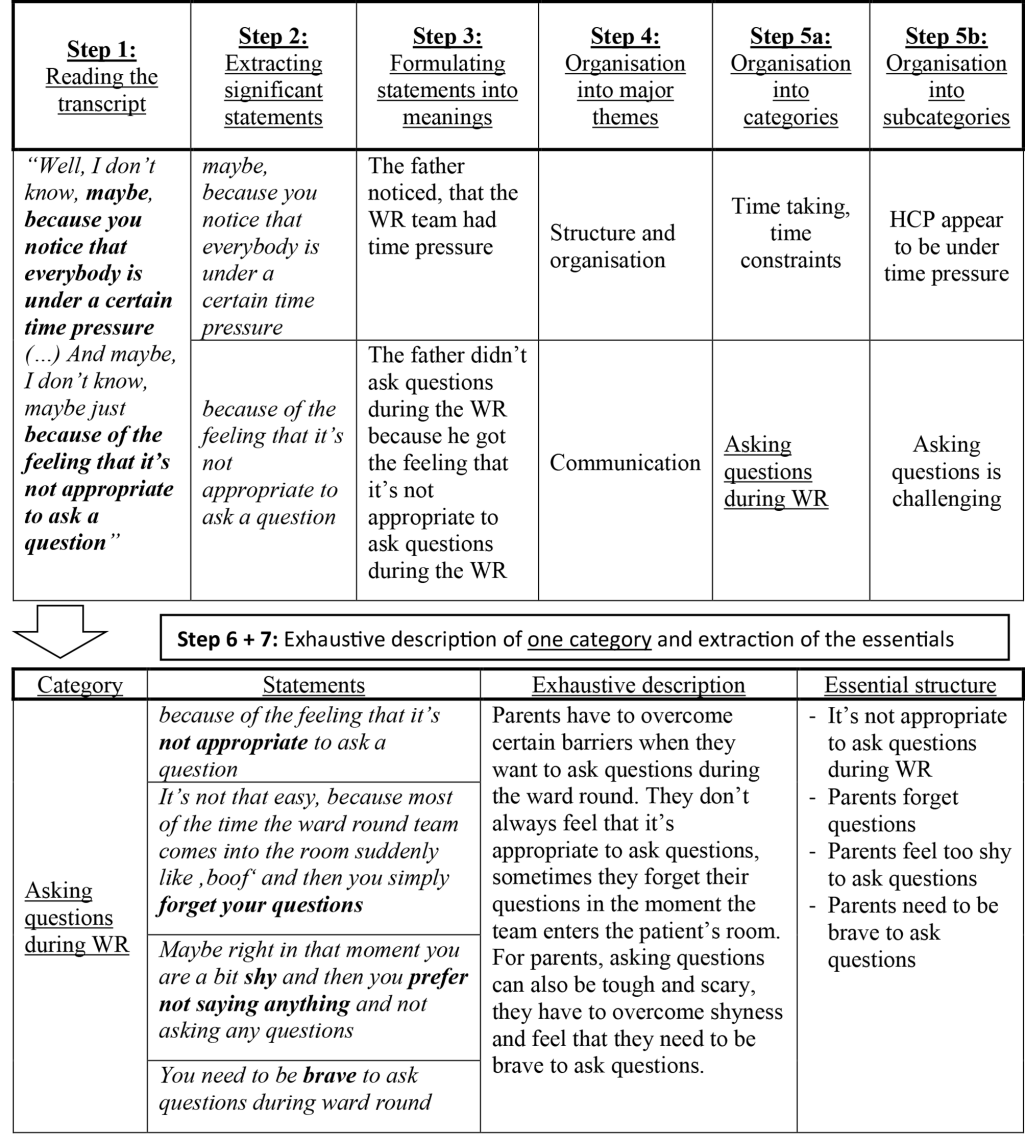
Example of the analysing progress from interview D (Father). Answer to the question: “Could you explain in more detail why you restrained yourself during ward round?”

Transcription was done by LB and checked for accuracy by UM following predefined transcription rules [24]. Transcripts were reviewed quote by quote and analysed using the framework by Colaizzi with respect to phenomena experienced during WR. This rigorous method is broadly accepted as an efficient tool to extract relevant information from interviews or written text [17] and follows seven defined steps (an example of the analysis process is given in Fig. 1): (1) Each interview transcript was read repeatedly (2) Significant statements were extracted and collected in a separate analysing Table 3. Statements were then rephrased into formulated meanings and 4. organised into clusters of **major themes** (MT). 5. Within these MT, formulated meanings were sorted into **categories** and **subcategories**. 6. For each interview all formulated meanings within the categories were condensed into an essential structure. 7. Essential structures from all interviews were put together into a table to enable comparison between different interviews. Step 1 was done by three researchers (LB,LG, JS). Subsequent steps were initially done by LB and then validated by team discussions until reaching consensus and confirmability (LB, LG and JS). For this purpose, the research team met on a weekly basis during the extraction process.

## Results

A total of 13 interviewees were recruited: Three medical doctors, three nurses, three parents, and four patients. Patients were between 11 and 18 years old, HCP had between one and 31 years of work experience.

The structured and stepwise analysis of the interviews resulted in a set of three major themes (MT): (1) Structure and organisation; (2) Communication and (3) Education with a total of 23 corresponding categories and additional subcategories (Table 2).

**Table 2:** All three MT (= major themes) with their categories and subcategories. The two right columns indicate in how many interviews with HCP (= health care professionals) and PP (= parent/patient) the category appeared. For better overview, categories that have appeared more frequently are highlighted in bold (≥ 4 (HCP) or ≥ 5 (PP)).

Categories	Subcategories		
**MT 1: Structure and Organisation**		**HCP**	**PP**
Preparation	knowing patient/medical history; all information available; WR-team complete?	**4/6**	**6/7**
Structure	fixed starting time; time management; agreed definition; rules; prioritising topics; structured WR- guide; clarify roles of HCP for families; decision & result	**6/6**	**6/7**
Control and revision of current status	current status of patient; check therapy plan; red flags/things missed?	3/6	1/7
Making a plan	next steps; plan for the day; distribution of tasks, day of discharge; prognosis	**4/6**	**6/7**
Patient’s daily assessment	physical examination; patient’s current wellbeing; course of disease	3/6	**6/7**
Freedom from interruption	concentration and discipline; focus; no interruptions; no mobiles;	**6/6**	1/7
The right place / setting	patient’s privacy; fellow patient as a passive listener; adjusting the place to the content of conversation	1/6	3/7
Time taking, Time constraints	time for detail; individual need of time; time for complexity; well-considered decisions; time pressure	**4/6**	3/7
Team make up	avoid large groups (scary, impersonal, like a show, prevent families from asking sensitive questions); large groups give the feeling of being cared for/looked after; continuity of WR participants	3/6	**6/7**
**MT 2: Communication**		**HCP**	**PP**
Exchange of information	shared information between different HCP groups; objective information; balancing act between medical content and meaning for families	**4/6**	**7/7**
Joined thinking	interface between different HCP groups; coordination of doctor’s and nurse’s work; conjoining knowledge and work experience; bringing everyone to the same level of knowledge; avoiding misinformation through whispered rumours, creation of solutions; reach of consensus; shared-decision making	**6/6**	3/7
Explaining medical facts	information about side effects; understanding the illness; ignorance scares families; reducing worries; bedside-teaching	0/6	**6/7**
Emotional support	being looked after; being cared for; presence of doctors triggers positive, cared-for feelings for patients; WR motivates & supports families; WR gives a sense of security; WR offers space for addressing worries; shared decision-making offers security for HCP	**5/6**	**7/7**
Building relationships and trust	getting to know the HCP team; getting to know the patient; encountering the “person behind the doctor”; understanding doctor’s decision-making processes; building up trust; only possibility to meet doctors on wards; possibility for personal conversation	3/6	2/7
Multidisciplinary team = opportunity and challenge	the more diverse HCP, the more perspectives; different work routines make WR organisation complicated; hierarchies threaten good communication; WR needs a decision-maker; parents feel dependent on the WR team	**6/6**	3/7
A respectful working atmosphere	acceptance of other opinions; space for controversy/critique; respectful working together; feel-good work climate; honesty; empathy; keeping promises; professionality; reliability; showing interest in the WR; all participants need to know each other	**4/6**	**6/7**
Communication on eye-level	finding a common communication level; adaption of communication to the level of knowledge; absence of hierarchies; communication suitable for children; reflection of communication; communication strategy; direct sharing of information; non-verbal communication	3/6	**5/7**
Nursing staff in ward rounds	nursing staff supports parents; nurses are communication mediators between families and doctors; involvement of nursing staff in bedside WR; information flow between nursing staff and medical doctors, time constraints for nursing staff	**4/6**	4/7
The parent as the patient’s representative	parents comfort their children; parents translate what doctors say for children; the parent as the child’s representative; parents speak up for their children’s needs	0/6	**5/7**
Asking questions during WR	HCP need to allow/offer space for questions; asking questions is challenging; the right atmosphere to ask questions; making it easier for parents/children to ask questions; health care team as the only reliable/good source of information	2/6	3/7
**MT 3: Education**		**HCP**	**PP**
How is the WR taught?	lack of WR-training during medical/nursing school; lack of briefing for job beginners; WR participation only during internships	**4/6**	0/7
How do participants learn about WR?	learning by making mistakes; learning by doing; learning by copying; higher position enables more active participation	**6/6**	0/7
WR as a learning process	over time, WR becomes less stressful; WR becomes familiar; understanding the purpose of WR; getting a better overview; becoming more self-confident; learning to be more critical; becoming part of the team; gaining knowledge and experience	3/6	**6/7**

**Table 3:** Suggestions for successful ward rounds (WR) derived from the interviews

Structure and organisation	
Agree on a starting time - with all stakeholders involved	
Use the best place available and avoid any distraction	
Prepare an agenda/ a structure or use a guideline	
Agree on aims for the WR	
**Communication**	
Create a respectful communication atmosphere	
Use layperson language when talking to patients	
Relationship building is necessary and not a waste of time	
Always leave room for questions	
Respect the families’ emotional state during the WR	
**Education**	
Prepare patients and families before their first WR to reduce anxiety	
Include medical and nursing students and teach them Create an atmosphere of appreciation– especially whilst teaching in WR	

### MT 1: structure and Organisation

Interviewees stressed important organisational requirements for a good WR. While families put emphasis on “patients’ daily assessment” and “team make up”, HCP focused on “freedom from interruption” and “time taking”. Categories that were mentioned most in both HCP and families’ interviews were “preparation”, the “need of a clear structure” and “making a plan” as one of the main requirements of a WR.

“Making a plan” came out to be the most important topic for both HCP and families. A nurse stated: *“In the end of the ward round, I want to have a to-do-list (…). And likewise, I want to be certain that the doctors also have their to-do-list”* (HCP 2). The WR helps structuring the day by planning next steps and distributing tasks. For families, the planning for the subsequent days also was an important part of WR. Information about the course of the next days and the day of hospital discharge was of utmost relevance for families in this study.

Interviewees criticised that WR sometimes proceeded in an unstructured manner. *“You sometimes don’t notice certain things, you just jump to the obviously pathological results (…) and then you overlook important information”*, a medical doctor explained (HCP3). Not having a clear structure of the WR was also recognised as a time waster. Some interviewees suggested to follow a standardised guideline to give structure by defining the order of topics and prioritising them.

In order to conduct a well prepared, structured WR, HCP pleaded to eliminate any distractions during WR. *“There are a lot of interruptive elements, like telephones or people rushing in”* (HCP6) and *“Every time someone else gets a telephone call, you lose track”* (HCP3). This was perceived as *“time-consuming”* (HCP6) and *“less efficient”* (HCP1). Still, they admitted that it is not possible to achieve complete freedom from interruption: *“When my patient suddenly becomes hypotensive, I have to seek for advice from the doctor’s room, no matter if it’s ward round at the moment or not”* (HCP1).

The appropriate length of WR was a controversy in the interviews. While parents and patients felt that WR was too short, medical doctors by contrast generally underscored their ‘time pressure’. Medical doctors expressed the wish that WR should be (more) effective and less time consuming for them. HCP nevertheless admitted that it was important planning enough time for questions. For HCPs, the most important issue was to keep enough time to thoroughly consider choices. They also emphasised that due to time pressure, decisions sometimes get postponed to the next day.

Interviewees described both advantages and challenges of interprofessional teamwork in the WR. While WR was perceived helpful to coordinate different work routines, these different work routines also made organisation of the WR difficult. “*Especially for nurses, planning time for ward round is more complicated, because they have to keep up with their work. They have a different time schedule than medical doctors.*“ (HCP 3).

Concerning team make up, most interviewees preferred a smaller WR team. A resident underscored, large WR teams turn the WR into a *“show event”*, which was *“absurd”* (HCP4). A patient said: *“When the senior doctor conducts the ward round, it feels as if basically everyone from the team attends. (…) You then feel like being under siege, when everyone is standing around looking at you.”* (PP4). Other interviewees indicated that large groups were ‘impersonal’. They made families feel uncomfortable about asking questions and stopped them from talking about sensitive topics. In addition, one patient stated that large teams at least make patients feel well looked after.

### MT 2: communication

Regardless of their role in the team, ‘communication’ played a critical part during WR for all interviewees. In particular, two different aspects of communication were underscored: First, exchanging information, and second, building relationships and providing emotional support.

Interviewees of all four groups agreed that “exchange of information” is one substantial function of WR: *“On the one hand, it’s just all about the trivial transfer of knowledge. (…) Objective knowledge, that’s what ward round is all about.”* (HCP 4) During WR, participants are brought to the same level of knowledge. For HCP particularly, exchanging knowledge initiated thinking processes and provided the basis for cooperative problem solving. They pointed out that WR also served to evaluate the patients’ current health status, to revise therapy plans, and to reveal errors before they happen.

Handing information from HCP to families was also brought up in the interviews. While HCP described information as “*objective*” (HCP4), for families every information they received during WR came together with a meaning or set off an emotion. *“The ward round team has to manage the balancing act between: We have to get the ward round done quickly and somehow ‘just’ communicate medical knowledge, while always keeping in mind what the things actually mean for the families”* (HCP4). Sometimes the fear of unfavourable answers would even keep parents from asking questions during WR: “*What if during ward round you ask ‘Is my child going to recover?’, and the doctor says ‘no’? I guess, that would not happen, but still.*“ (PP2). Families on the other hand also emphasised that explanations of medical facts and getting information during WR helped them cope with the situation.

Besides information exchange, “emotional support” was mentioned repeatedly within this MT. Parents explained, WR helped them to cope with the stressful situation. Knowing their children were looked after comforted them: *“Because you have to face this situation (…) and the ward round can support you doing that by saying: This is reality now and we do our best so that everything will be fine again”* (PP2). Patients underlined that WR made them feel valued and looked after. This is illustrated by a quote from a patient’s interview: *“It’s just nice that you are still seen regularly by the doctors, just so they can ask you: How are you? Are you okay?”* (PP4). HCP felt more secure with regard to solving problems by sharing responsibility with their colleagues.

Nurses and Patients brought up the WR function of “building relationship” and “providing trust”. Creating a bond between HCP and patients rendered the process of therapy and associated decisions more personal. Patients flagged up the importance of getting to know the medical doctor’s ‘character’. It helped them understand the medical doctor’s decision-making process. An adolescent patient explained: *“As a patient, you (…) allow them to take control over your therapy and everything else. So, you put a lot of trust in them without knowing who the person is. Who is this person deciding about my health in a context where I can’t decide on my own?”* (PP5).

Parents and patients highlighted they would have preferred understandable (layperson) language during WR. In addition, parents and families underscored the need for HCP to adjust communication to their emotional situation. One patient (PP5) put special emphasis on non-verbal communication. She described a situation experienced during WR: *“To me, it looked like they were not sure what the results of the sample would be, or rather actually I already figured out that - when it takes so long to get the results - there’s a reason for that (…). The ward round team didn’t convey any particular uncertainty, they just said: ‘we have to examine that further’. But when you think about it (…), you will quite easily come to the conclusion that something must be abnormal”* (PP5).

All interviewees pleaded for a “respectful working atmosphere” during WR with “Communication at eye-level”. They wanted the WR participants to be appreciative, with active listening-skills.

Nurses stated that, within WR discussions between HCP, there were no doctor-nurse-hierarchies. Every team member was equal. HCP therefore felt comfortable with openly expressing their own opinion. During WR at the bedside though, doctors and families sensed the existence of hierarchies, especially when senior doctors attended. These WR were “*less familiar*” and not as “*relaxed*” (PP5). A mother explained: “*I would like the junior doctors to have more responsibility within the ward round conversation (…) as they are the ones actually seeing and treating the kids on a daily basis*” (PP1). Some HCP on the other hand still underlined the benefit of a “senior WR participant”, who feels responsible for decision-making.

Interviewees of all four groups support the notion to include experts for psychological and/or social care into the WR. These experts could support the teams’ decision making and take care of questions regarding non-medical-topics. Also, they could contribute a new perspective to the ward round discussion. As a doctor stated: *“If we had the psychosocial team join the ward round, they could also get an impression [of the patient and the family], indeed, they get a completely different impression than the doctor”* (HCP 3).

### MT 3: education

All HCP stated that they never learned how to conduct a WR in nursing or medical school. Once they started their career, they learned about WR and their tasks by attending and then simply copying what other colleagues were doing. No HCP mentioned a WR training during their continuing education.

The majority of interviewees described a change of attitudes and skills over time, and reflected the impact on their own WR behaviour. HCP underscored the increasing experience, which in turn boosted their self-confidence. As a consequence, they participated more actively during WR and expressed their opinions more openly. Parents underlined that initially they were not brave enough to criticise medical staff during WR. In the beginning of the disease, they would only receive information passively, but seldom actively communicated their own opinions and fears. “*I don’t think the ward round has changed over time. It’s more that throughout the duration of treatment, things have become clearer to me. (…) You simply have to work on yourself and actively contribute to the ward round*” (PP6). During the course of the oncological treatment and whilst acquiring more knowledge, they became more critical and occasionally questioned medical decisions. Additionally, parents asked for a guideline to make the WR more transparent in the beginning of the treatment: *“I would like to have an information leaflet for families about the ward round before the first stay in hospital. That information could explain: what is the ward round, what parts does it consist of? And what should it clarify?”* (PP1). At first, parents often didn’t understand the purpose of WR and if they were allowed to ask questions.

When patients started their cancer treatment, the WR scared them as something special and alarming: *“In the beginning it’s always like, (…) really terrifying anxieties: ‘Oh my god, the doctors are coming, something really important must be going on’. And then over time you simply learn: okay, they just want to look how you are and tell you the next steps and not tell you the next dreadful news”* (PP4). After attending a few rounds, WR became a normal daily routine.

## Discussion

WR is an essential clinical routine for teams in the hospital setting. In paediatric oncology, the average length of treatment process is 8 months. During in-patient periods, patients experience WR on a daily basis. During this time, patients and their families therefore gain experience in communicating with different HCP and get to know the hospital structure. The long-lasting treatment process and the daily rhythm of WR makes it even more important to gain a better understanding of paediatric oncology WR. Similarly, these prerequisites turn the area of paediatric oncology into the ideal field for a study about WR experience and WR expectations. Additionally, involvement of patients into shared-decision-making is more complex and demands special consideration [13]. To the best of our knowledge, this is the first qualitative study analysing the function of WR in paediatric oncology through the lens of several relevant stakeholders. Previous studies have examined certain aspects of the WR, such as setting, duration, different participants roles and functions, the use of checklists and communication between team members [8].

### MT1: structure and organisation

Research agrees that preparation, elimination of distractions and a systematic structure are of great importance for conducting an effective WR [1]. Despite these insights, interviewees in our study underlined that they were still missing a good preparation and clear structure. Medical doctors raised concerns about the risk of missing warning signs, when not having a defined structure.

In the literature, the optimal WR team composition and size is not yet defined and often varies [25]. Our data indicate that WR team composition and size is controversial and should be individually tailored to the situation. Families pleaded for smaller teams. They explained, large WR groups intimidated them and made them feel less involved. HCP in contrast preferred a multidisciplinary team involving different medical specialities. Research indicates that multidisciplinary WR achieve improved teamwork, better consideration of patient’s needs and also greater patient involvement, but this research did not include the patients’ view[26]. Our data shows that team size is a double-edged sword, as bigger teams can be overwhelming for patients and parents.

Leadership was not an important factor for successful WR in this study. Having a consultant attending the WR was even criticised by families and junior doctors. Conversations were then perceived as less open and rather strained. This stands in contrast to Merriman and Freeth’s findings, who observed consultants lead WR and pointed out that in their setting (adult critical care units), “good leadership styles are the core element of a well-coordinated and integrated provision of care”. Additionally, they stated that leadership does not depend on the education degree and “positional authority”, but on clinical expertise and experience[9]. This illustrates how multifaceted WR are in different clinical settings and underscores the need for research.

Considering time, researchers have tried to define the optimal length of a WR in the past. According to this research in the setting of acute general internal medicine they came to an optimal WR time of 10 to 14 min per patient regarding best “quality and safety at the point of care” [27]. Another recent study concludes that more time at the bedside is not associated with a positive effect on the patient’s experience in WR. More important than time alone was the quality of the time spent at the bedside [28]. Interviewees of the present study indeed had different concerns regarding WR length. While families experienced the WR as too short and hectic, for HCP it always felt too long. Interviewees summarised, WR participants have to plan enough time for details and adapt the length of stay to the patient’s current situation. Taking together, time is critical during WR – HCP should be empowered to provide ‘quality time’ for the patients while in parallel families should be encouraged to actively make WR a helpful time regarding their needs.

### MT 2: communication

In this study, communication appeared in the context of two very different aspects: On the one hand, WR was described as a tool for exchanging information. In the literature, this is described as the most important function of the WR [29]. The other aspect concerned the emotional component of communication. Of note, 12 out of 13 interviewees of our study defined “emotional support” as an important WR function beyond mere information exchange.

Research shows that errors in the treatment of hospitalised children lead to prolonged hospital stays and higher mortality [30, 31]. Sharma et al.[32] identified the WR as a potential source of errors: Failure in communication can cause lost, misunderstood or unarticulated information. By contrast, improved communication within the team and between medical doctors and patients can reduce errors [33]. Poor communication can be attributed to the use of medical jargon, apparent time constraints and the lack of empathetic change of perspective [4]. Our data contributes that structural considerations should be added to the list of aspects needed for successful communication. Of note, there are global attempts to implement WR communication guidelines, to help reducing errors, and improve communication in WR [32, 34]. However, this has not yet become standard practice, and such guidelines are limited to medical subspecialities.

It is well-known that in paediatric oncology, looking after families’ emotional concerns can prevent noncompliance [35]. Research shows that parents are encouraged by seeing many different people care for their children [36]. Additionally, collecting information comforts parents of children with cancer [37]. It is a coping mechanism helping people in stressful situations by reducing uncertainty and regaining a sense of control [38, 39]. A recent study exploring parents’ perceptions of WR highlighted that parents highly value WR primarily for the opportunity it offers to collaborate with the clinical team and to ask questions [10]. Still, none of these studies defined “emotional support” as a function of the WR. If this was an academically recognised WR function, participants could become aware of this aspect and better exploit it.

In this study, only patients and nurses described the WR as an opportunity to build and strengthen relationships, while neither parents nor medical doctors mentioned this. Patients explained that knowing their medical doctors helps them understand decisions. Decision-making is a complex process and is influenced by personal attitudes, values and beliefs [40]. Understanding how medical decisions are made helps patients to accept and support the decision and improves their cooperation [12]. Another study describes how adolescents and young adults with cancer struggle with the large number of strangers surrounding them in the beginning of their treatment. They therefore develop strategies to get to know HCPs in order to decide “whether or not they could trust HCP” [41]. Similar to our results, this study concluded that opportunities to connect with patients are often missed by HCP, even though a good “connectedness” can reduce the risk behaviour of adolescents with cancer [41]. Simultaneously, patient’s WR experience depends on a good relationship to the WR team, as revealed by Cappleman et al. [42].

### MT3: education

Finally, our results indicate that neither professional team members nor families felt well prepared for WR. International research has already revealed this issue for medical education. While communication skills receive now greater regard in university education programmes, the WR is not standard part of most curricula [6]. Recently, research has explored new WR training programmes for healthcare staff [43, 44]. One medical school in Germany incorporated the WR into their curriculum on a trial basis. According to the results, students rated the training as authentic and felt better prepared for WR afterwards [45]. It is striking that most of the new WR training programmes are designed for medical students only, despite scientific agreement on teamwork and communication being significant elements of WR [11]. Research shows that interprofessional learning can improve both [46]. In our study, not only medical doctors, but also nursing staff pleaded for a better WR preparation during their education. For medical education this study therefore adds important information: WR needs to be taught interdisciplinary so that medical doctors and nursing staff (and possibly other professional groups) both learn how to conduct a WR as a team early on.

Patients of this study also unveiled that emotional preparation for WR was challenging. Initially, they were scared of WR, until they understood that it is a daily routine. This is consistent with Berkwitt and Grossman’s findings [47], who observed that in the beginning of their hospital stay, paediatric patients associate WR with negative feelings. It can therefore be assumed that healthcare staff could spare patients anxiety and insecurity by explaining the purpose of WR early on, which is also supported by other studies [48].

This study aimed to explore the detailed insight of different WR participants, in order to gain a better understanding of their experiences and attitudes towards WR. Important aspects have been revealed which should be implemented in a successful WR routine. Very importantly, the need for building relationships during WR and giving room for emotional support should be acknowledged by every professional WR participant. This study also stresses the need for interdisciplinary WR training in medical education, as well as preparing families for the WR when treatment starts (by a leaflet or consultation).

Our study has several strengths and limitations. First, our data represents the experience of staff and patients’ families of a single institution and took place at a German paediatric oncology ward. Experiences of members of WR at other (oncology) wards may differ and medical systems work differently in different countries. However, our study focused on a multidisciplinary team, patients with severe illnesses and their families, emotional and stressful situations, and relationships between different WR members. These aspects can be found in other wards beyond (paediatric) oncology. We therefore assume that our results have external validity to some extent. In this study, translating the interviews from German to English carries the risk to possibly miss linguistic and cultural nuances as well as ambiguity and multiple meanings or idiomatic expressions and colloquialisms. We therefor hired an experienced translator for the process and incorporated a proofreading step to review the text for accuracy and adherence to the intended meaning. In addition, considering whether participants received formal training for ward rounds helps them to contextualize their expertise, assess variations in practice, and also influences the mindset regarding the ward rounds and should have been more formally explored. Thirdly, one might argue the potential bias caused by coercion effects in this study. It is emphasized that it is impossible to fully exclude these coercion effects. For example, patients may feel obligated to the medical staff out of gratitude, while healthcare professionals may perceive hierarchical structures and be influenced by them. These implicit dependencies can influence the respondents’ statements and lead to result bias. Therefore, it is important to consider these potential biases and consider alternative approaches or methods to ensure objectivity and accuracy in future studies.

As we included only interviewees with German as a first language, our results cannot be generalised to non-native speakers. Further important aspects of the WR might have not been covered by our study, as their experience might be different. Language barriers are still a huge problem in healthcare [49]. Social, cultural and religious differences are additional obstacles in the treatment of paediatric patients [50]. As the WR is the central place of communication, these aspects should be addressed in future research with focus on both sides – patients/families as well as HCP.

Lastly, we included only a limited number of interviewees. On the one hand, within this small cohort, maximum diversity was attempted to achieve with representation of all major groups participating in WR, work-experience from one to 31 years, different genders and age variation. We stopped the recruitment of participants when we reached theoretical saturation [23]. Nevertheless, due to the limited number of participants, a representative analysis of participants’ characteristics, such as work experience or age, in relation to their attitudes towards WR was not possible.

The amount of scientific literature regarding WR is limited, and, to our knowledge, does not cover comparing experiences of all different stakeholders. Thus, this study adds a substantial contribution to the small but growing body of published data regarding WR in general and in paediatric oncology in particular.

## Conclusion

Our research showed that for stakeholders involved in in-patient paediatric cancer care, the WR has a bundle of different important functions, aims and potentials. The three MT that emerged from this study were “organisation and structure”, “communication” and “education”. It became apparent that there is a huge variety regarding expectations towards WR among different stakeholders. Nevertheless, all WR participants pleaded for a clear structure within the WR and better preparation. They suggested that the HCP team should organize the WR setting together, in terms of starting time, suitable place and elimination of disruptive elements. Also, they raised the wish to strengthen the multidisciplinary aspect of the WR by including additional clinic staff, like for example members of the psychosocial team or physiotherapists. Participants also expressed a need for improvement with focus on relationship building, emotional support and communication. Almost all participants were not trained (HCP) or prepared (patients/parents) for their first WR. Information for families explaining WR aims and functions might be helpful. HCP discussed the need of training programs during medical and nursing school, as well as regular inhouse training. The knowledge gathered in this study gives valuable impulses for a better understanding of a successful WR that meets the expectations of different stakeholders. It can also serve as a knowledge base for interdisciplinary WR training.

## Data Availability

The datasets of raw interview material generated and analysed during the current study are not publicly available due protection of vulnerable groups (minors) and the participants privacy, but are available from the corresponding author on reasonable request.

## List of Abbreviations

WR: Ward round
HCP: Health Care Professionals
PP: Patients/Parents
MHH: Medizinische Hochschule Hannover / Medical School of Hannover
MT: Major Theme
